# Multifocal and Recurrent Tonsillar Squamous Cell Carcinoma

**DOI:** 10.7759/cureus.67871

**Published:** 2024-08-26

**Authors:** Rehman Basharat, Ghassan Samara

**Affiliations:** 1 Otolaryngology, Stony Brook University, New York, USA

**Keywords:** squamous cell carcinoma (scc), oral cavity neoplasm, head and neck cancer surgery, oropharyngeal cancer, tonsillar cancer

## Abstract

The following study describes a complex clinical course of recurrent and multifocal squamous cell carcinoma (SCC) of the tonsil, involving both initial and subsequent malignancies over several years. The patient, a 54-year-old male with a history of tobacco use, first presented with SCC of the left tonsil, treated with tonsillectomy and neck dissection. Despite clear margins post-surgery, the patient developed SCC in the right tonsil two years later, requiring further surgical intervention and a comprehensive treatment approach. The disease then progressed to the base of the tongue and right larynx, necessitating a total laryngectomy and subtotal glossectomy. The report emphasizes the critical role of advanced imaging and surgical techniques, including robotic-assisted surgery, in managing such complex cases. Additionally, the case highlights the challenges of treating advanced oropharyngeal SCC, the importance of multidisciplinary management, and the need for consistent follow-up to monitor treatment efficacy and manage complications. The case underscores the complexity of SCC in the head and neck region and the necessity for tailored therapeutic strategies to improve patient outcomes.

## Introduction

Cutaneous squamous cell carcinoma, also referred to as squamous cell carcinoma (SCC) of the skin, is the second most prevalent type of skin cancer in the United States, with basal cell carcinoma being the only form that occurs more frequently. SCC commonly develops from a previous condition called actinic keratosis, which has the potential to become cancerous. While SCC can spread to other parts of the body, it is not as common as with other types of skin cancer [1]. The primary cause of cutaneous SCC is typically long-term exposure to UV radiation, but other factors like chronic exposure to carcinogens from cigarette tar can also play a role in its development. SCC can arise in persistent scars, extensive burns, or prolonged skin ulcers. High-risk variations of the human papillomavirus (HPV) are also a major contributing factor to the development of SCC, particularly in individuals with weakened immune systems. These individuals have a much greater chance of developing SCC in their lifetime, and the risk of cancer spreading to other parts of the body is higher in these cases [2,3]. From an epidemiological standpoint, SCC is very common, with over one million cases identified every year in the United States. This tumor tends to be more prevalent among the elderly, especially those aged 50 and above, and is slightly more common in men as well as individuals with fair skin and light-colored eyes [4,5]. SCC exhibits various subtypes histologically, such as squamous cell carcinoma in situ, Bowen disease, acantholytic/adenoid/pseudoglandular, clear cell, sarcomatoid/spindle cell, keratoacanthoma, and verrucous carcinoma. These tumors are identified by unusual keratinocytes with lots of pink cytoplasm, parakeratosis, connections between cells, and clumps of keratin [6]. The thickness of the lesion is an important factor in determining the risk of metastasis, with a thickness exceeding 4 mm linked to a greater risk. Immunohistochemical staining for cytokeratins 5/6/AE1/AE3 is highly valuable in difficult diagnostic scenarios, particularly with poorly differentiated SCC [6]. The subtype characterized by clefting around tumor cell nests results in a glandular appearance due to desmosomal disruption and is known as the acantholytic/adenoid/pseudoglandular subtype. This characteristic sets it apart from genuine glandular tumors like adenosquamous carcinoma, which usually do not show carcinoembryonic antigen (CEA) staining [6]. The subtype of sarcomatoid/spindle cell is characterized by spindle-shaped keratinocytes with pleomorphic nuclei and many mitotic figures, making it difficult to distinguish from atypical fibroxanthoma, which typically does not show positive results for p63 and p40, unlike sarcomatoid SCC [7]. Distinguishing desmoplastic SCC from desmoplastic melanoma is necessary, with immunostaining for p63 and S100 being helpful for diagnosis due to the similar features such as dense collagenous stroma and perineural invasion [7]. This case report focuses on a patient with a complex history of recurrent and multifocal SCC, detailing the clinical course and treatment strategies undertaken over several years. The patient's case underscores the critical role of multidisciplinary management and highlights the challenges associated with advanced SCC in the head and neck region.

## Case presentation

**Figure 1:**
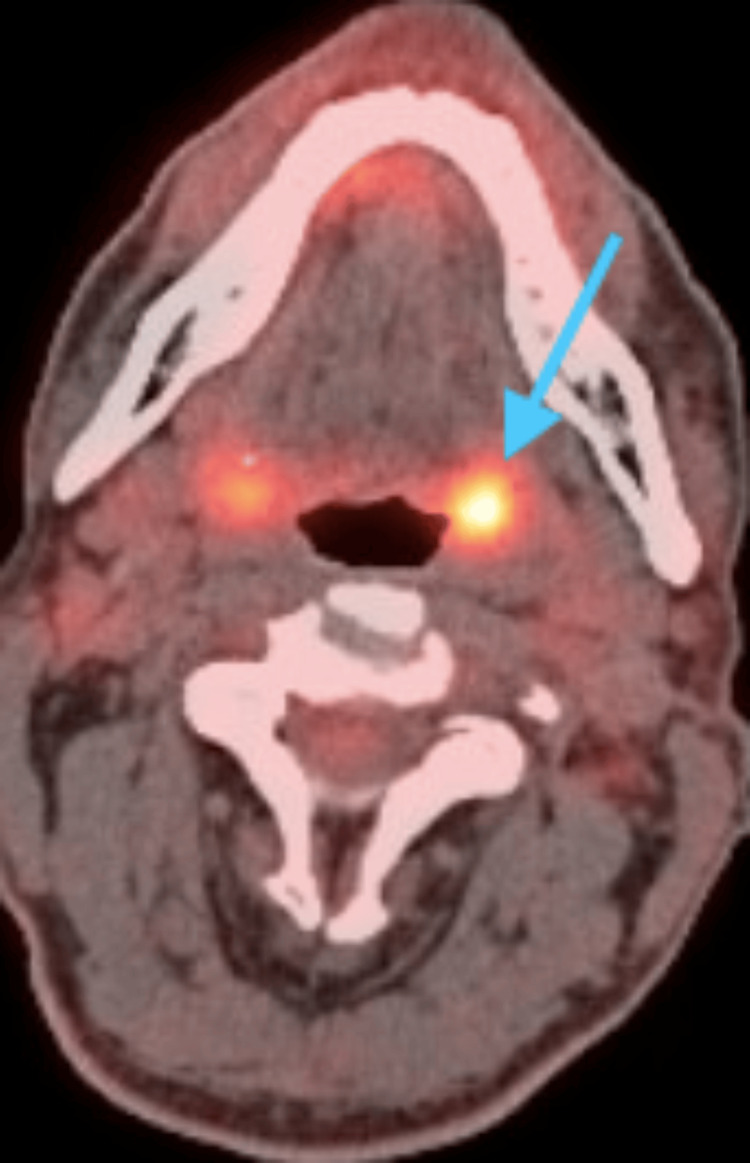
Axial PET scan showing hypermetabolic activity in the left tonsil with a standardized uptake value of 9.7, indicating malignancy. PET: positron emission tomography

In 2015, a 54-year-old male with a history of tobacco use presented with SCC of the left tonsil, staged as pT1N2 p16+. A positron emission tomography (PET) scan confirmed the presence of the malignancy, necessitating surgical intervention (Figure 1). The patient underwent a left radical tonsillectomy, which included partial palatectomy, partial phalangectomy, and partial glossectomy, performed using a robotic-assisted surgical system. The operation commenced with general endotracheal anesthesia, followed by the placement of Aquaplast splints to protect the teeth. A FKWO retractor was positioned in the oral cavity, and a 2-0 silk suture was used as a retraction stitch on the tongue. The resection began with incisions through the anterior and posterior pillars of the soft palate, extending through the pharyngeal constrictor musculature. A portion of the pharynx was resected with a vertical incision, and the resection continued anteriorly near the retromolar trigone. During the surgery, large vessels were encountered and were clipped using multiple surgical clips, while smaller vessels were cauterized. The tongue base was resected with a vertical midline incision, followed by a horizontal incision along the retractor margins, extending to the vallecula where a mucus retention cyst was drained. The wound closure involved a running V-Loc suture, with some areas left open and covered with Tisseel to facilitate healing. To perform the modified radical neck dissection, a cut was made from the neck, going through the platysma muscle. Subplatysmal flaps were carefully elevated in different directions: anterior-superiorly, posterolaterally, and inferiorly. The superficial layer of the deep cervical fascia was incised over the sternocleidomastoid muscle and in the midline and then reflected laterally. The dissection continued until reaching the hyoid bone, and then the remaining tissue was moved to the side, revealing the common carotid artery and vagus nerve. Complications occurred when the vagus nerve was discovered surrounded by dense desmoplastic tissue near the tumor during the skeletonization of the jugular vein. While dissecting, the hypoglossal nerve was identified posteriorly and superiorly, also being surrounded by desmoplastic tissue. Further examination uncovered two solid lymph nodes close to the base of the skull and the transverse process of C2, requiring that they be taken out. Unfortunately, both the jugular vein and the vagus nerve, and potentially the hypoglossal nerve, were encased within the dense fibrous tumor mass. The spinal accessory nerve was identified at both Erb's point and the skull base, and it was carefully freed from the tumor. The phrenic nerve was also identified and preserved during the procedure. Once all necessary structures were freed or removed, the fibrous attachments and cervical rootlets were divided, and the tumor specimen was excised from the neck. The surgical site was thoroughly irrigated and inspected for hemostasis, which was successfully achieved. The surgical wound was closed in layers, first with the platysma being sutured over a drain, followed by the skin closure, and finally, a dermal adhesive was applied to seal the wound. Post-operatively, the patient developed dysphagia which was treated through therapy.

**Figure 2:**
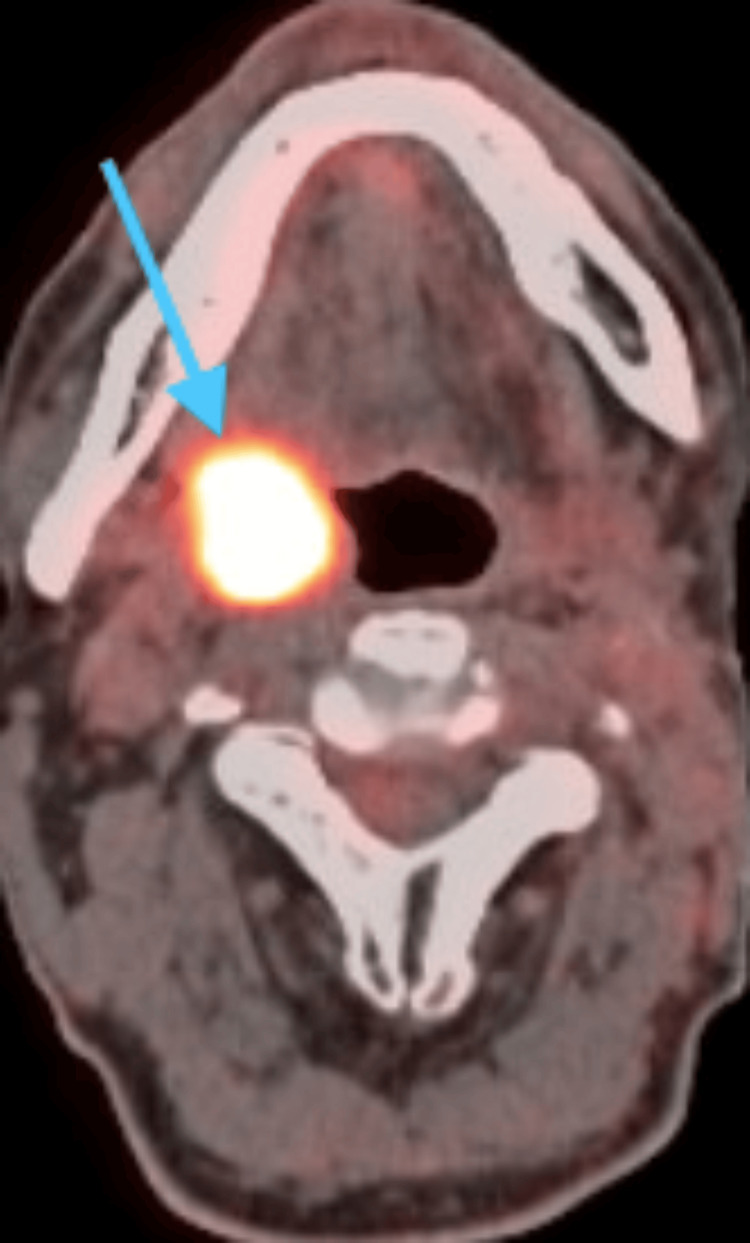
Axial PET scan showing hypermetabolic activity in the right tonsil with a standardized uptake value of 20.3, indicating malignancy. PET: positron emission tomography

In 2017, the patient then developed SCC in the right tonsil, staged as pT3N0 p16+, detected during routine follow-up (Figure 2). The patient underwent a transoral robotic radical tonsillectomy, which included partial palatectomy, partial pharyngectomy, and partial base of tongue resection. Additionally, a right modified radical neck dissection was performed, preserving key structures such as the spinal accessory nerve, sternocleidomastoid muscle, and internal jugular vein. Under general anesthesia, the patient was intubated nasally. The FKWO retractor facilitated exposure, and a mouth gag was used. Initial resection of the right tonsil utilized electrocautery. The surgical team then used the da Vinci robotic system for precise dissection, focusing on minimizing damage to surrounding structures. The tumor's friability complicated the resection, leading to rupture and requiring additional tissue removal, particularly from the pharyngeal margin, which was challenging to clear. Margins were repeatedly assessed, and further excision was performed until negative margins were achieved. The neck dissection involved a J-shaped incision, with careful dissection and preservation of the external and internal jugular veins, carotid artery, and vagus nerve. The submandibular gland and level 1B lymph nodes were resected, and the lingual nerve was preserved. Dissection proceeded to levels 1-5, with lymph nodes sent for pathological examination. Post-operatively, the patient received 60 Gray of radiation therapy delivered in 30 fractions (sessions), with a dosage of 2 Gray per session. Several months later, the patient developed respiratory insufficiency and required a tracheostomy.

**Figure 3:**
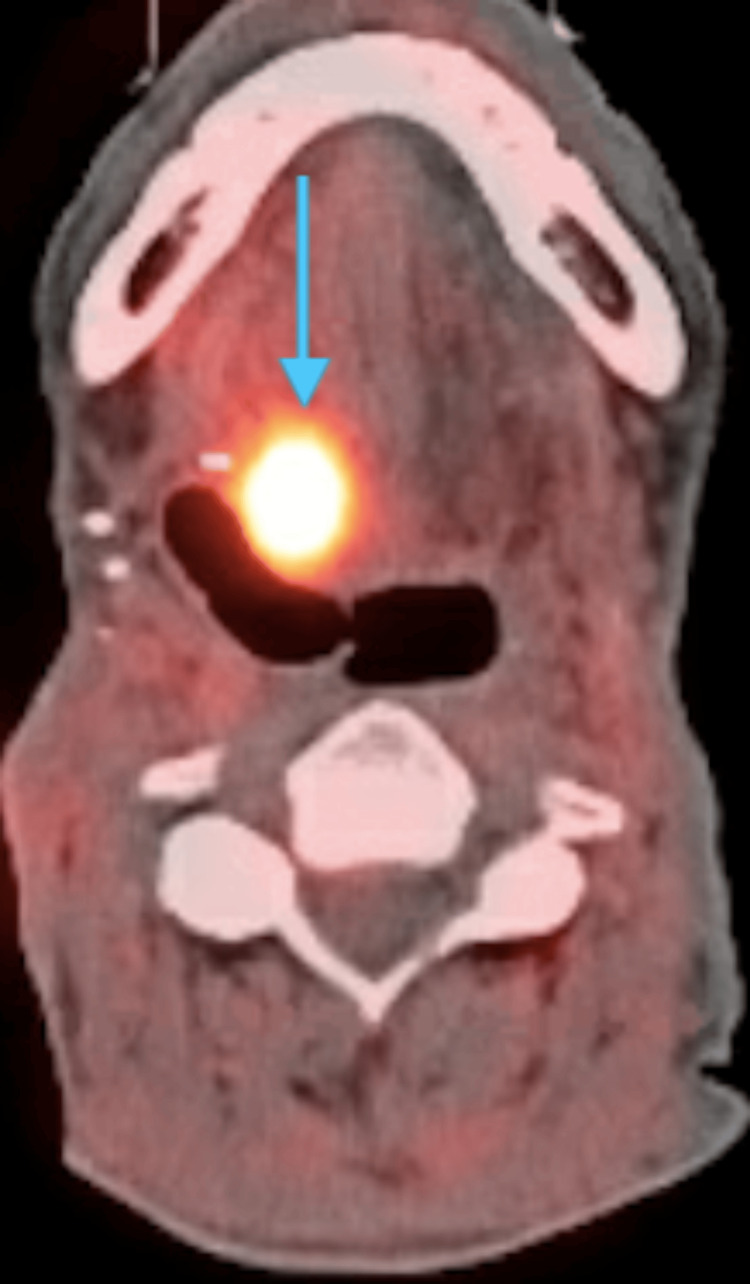
Axial PET scan with an abnormal hypermetabolic lesion in the sublingual region to the right of midline with a standardized uptake value of 15.6, indicating malignancy. PET: positron emission tomography

Then, in 2018, the patient's condition had progressed to advanced SCC involving the base of the tongue and right larynx, extending across the midline (Figure 3). This severe advancement resulted in aphonia, necessitating a total laryngectomy and subtotal glossectomy for comprehensive disease management. Concurrently, a percutaneous endoscopic gastrostomy (PEG) tube was placed to facilitate feeding given the extensive nature of the resections. The surgical approach included enhancing the previously made tracheostomy area and reopening part of an earlier apron incision from prior bilateral neck dissections. The surgery entailed meticulous dissection to ensure clear margins around the trachea and the base of the tongue, preserving as much normal tissue as possible while achieving oncologic control. Another physician then prepared vessels for a free flap reconstruction post-resection. After each surgery, the patient was prescribed acetaminophen at a dosage of 1000 mg every six hours for pain management. Additionally, the patient received a prophylactic antibiotic, amoxicillin, at a dosage of 500 mg every eight hours for seven days to reduce the risk of infection. The patient's labs were also noted pre-operatively and post-operatively to monitor recovery (Table 1). 

**Table 1:** Pre-operative and post-operative blood test results for a patient with tonsillar SCC over three years (2015, 2017, 2018). Reference ranges are provided for comparison. SCC: squamous cell carcinoma; WBC count: white blood cell count; RBC count: red blood cell count; MCV: mean corpuscular volume; MCH: mean corpuscular hemoglobin; MCHC: mean corpuscular hemoglobin concentration

	2015		2017		2018		Reference range
	Pre-op	Post-op	Pre-op	Post-op	Pre-op	Post-op	
WBC count	14.11	7.47	7.27	14.0	5.45	4.09	4.5-11.0x10^9^/L
RBC count	4.17	4.37	4.77	3.91	3.61	3.36	4.7-6.1 million cells/µL
Hemoglobin	13.2	13.7	14.9	12.5	10.2	9.5	13.8-17.2 g/dL
Hematocrit	39.7	41.7	47.1	37.8	32.3	31.1	40.7-50.3%
MCV	95.2	95.4	98.7	96.7	89.5	92.6	80-100 fL
MCH	31.7	31.4	31.2	32	28.3	28.3	27-31 pg/cell
MCHC	33.2	32.9	31.6	32.4	31.6	30.5	32-36 g/dL

## Discussion

Tonsillar SCC represents a considerable portion of oropharyngeal cancers, accounting for around 23.1% of tumors in this area, with a rate of 8.4 cases per 100,000 people [8]. The assessment of tonsillar SCC requires thorough imaging and endoscopic evaluations to properly stage the disease and help plan treatment. While CT scans are essential for assessing the size of the primary tumor and identifying nodal and pulmonary metastases, contrast-enhanced MRI is preferred for its exceptional ability to distinguish soft tissues. PET-CT is important for challenging cases and post-treatment monitoring [9]. It is highly advised to undergo endoscopic evaluation while under anesthesia for all suspected cases, as it allows for thorough assessment, biopsy, and surgical planning, as well as ruling out secondary malignancies in the upper aerodigestive tract [10]. Fine-needle aspiration biopsy (FNA) can be utilized for patients who are not suitable for surgery [11]. Surgery is the main treatment for tonsil SCC in the early stages, commonly using transoral robotic surgery (TORS) and transoral laser microsurgery (TLM). These methods are preferred because they result in decreased operating times, shorter hospital stays, and enhanced recovery of swallowing abilities when compared to traditional open techniques. Nevertheless, both methods have possible drawbacks, especially in the case of TLM. Histological margin evaluation can be complicated when using TLM because the tumor is removed in fragments [10]. For initial T3 tumors, TORS or TLM may still be possible, but more advanced T4 cancers typically need chemoradiotherapy, as extensive surgery would result in significant complications and complex reconstruction difficulties. Chemoradiotherapy remains the usual treatment for advanced tonsillar SCC, usually including cisplatin-based treatments. When cisplatin cannot be used, other options like cetuximab are taken into account. Due to the high frequency of nodal disease in tonsillar SCC, a preventive neck dissection that usually includes levels II-IV is frequently advised for managing and reducing the chance of metastasis [12]. Radiotherapy is successful for early-stage tumors, with one-sided radiotherapy usually enough for non-lateralized cancers, while radiotherapy on both sides is recommended for cases with nodal involvement on the opposite side to reduce recurrence risks [13]. Possible alternative diagnoses for tonsillar SCC may include actinic keratosis, erythroplasia, lichen planus, leukoplakia, lichenoid lesions, oral candidiasis, tonsillitis, and traumatic lesions. Severe complications can arise from tonsillar SCC and its treatment. If not treated, the tumor can spread to nearby structures like the lateral pterygoid muscle, pterygoid plates, lateral nasopharynx, and skull base or encase the carotid artery, causing dangerous issues like carotid blow-out [8]. Methods of treatment like TORS and radiotherapy come with potential complications such as postoperative pain, difficulty swallowing, mucositis, tissue scarring, decreased saliva production, infections, and osteoradionecrosis, all of which can greatly affect the patient's well-being [14].

## Conclusions

Tonsillar SCC is a significant and complex form of oropharyngeal cancer. The successful management of tonsillar SCC requires a multidisciplinary approach that includes advanced imaging, precise surgical techniques, and comprehensive oncological therapies. This case highlights the importance of early detection, thorough staging, and the need for a tailored treatment approach to minimize complications and improve patient prognosis. The high incidence of nodal involvement in tonsillar SCC further emphasizes the necessity for elective neck dissection and radiation therapy in managing the disease. Additionally, addressing potential complications and accurately differentiating SCC from other conditions are crucial steps in ensuring appropriate and effective treatment. Consistent follow-up care is essential for monitoring treatment efficacy, detecting recurrences early, and managing any long-term complications that may arise from treatment. Regular follow-ups enable timely interventions, which are critical in maintaining patient quality of life and enhancing survival rates.
